# Mitochondrial Myopathy, Lactic Acidosis, and Stroke-Like Episodes Combined with Diabetes: A Case Report

**DOI:** 10.2174/0118715303419754251125093050

**Published:** 2026-01-15

**Authors:** Shumin Zhou, Yongjun Tian, Fengying Liu, EnZhu Wu, Xiaodan Feng

**Affiliations:** 1 Department of General Internal Medicine, Guangzhou Development District Hospital, Guangzhou, 510700, China;; 2 Department of Neurology, Guangzhou Development District Hospital, Guangzhou, 510700, China;; 3 Department of Radiology, Guangzhou Development District Hospital, Guangzhou, 510700, China

**Keywords:** Mitochondrial diabetes, mitochondrial encephalomyopathy, mitochondrial DNA, hereditary disorders, diabetic ketoacidosis, genetic screening

## Abstract

**Introduction:**

Mitochondrial diseases refer to a group of hereditary disorders involving damage to high-energy-consuming tissues, such as muscles, nerves, and the heart. Mitochondrial DNA (mtDNA) mutations account for most cases, but the timely identification and treatment of these conditions remain challenging.

**Case Presentation:**

This report describes a case of a 36-year-old male patient who was diagnosed with diabetes in 2017 and subsequently experienced recurrent diabetic ketoacidosis and seizures. On May 20, 2022, he presented with cognitive impairment, unsteady gait, and an elevated blood lactate level. Brain MRI and mitochondrial gene sequencing on peripheral blood cells revealed destructive neuronal lesions and a mutation of m.3243A>G in the *MT-TL1* gene with a ratio of 6.04%, which supported the diagnosis of mitochondrial encephalomyopathy associated with lactic acidosis and stroke-like episodes (MELAS) and mitochondrial diabetes mellitus (MDM). Treatment with insulin, fluid replacement, ketoacidosis correction, diazepam, and phenobarbital relieved most symptoms. However, his blood glucose was poorly controlled. Four months after discharge, the patient suffered a relapse. Although therapies to combat infection, reduce blood glucose, and correct ketoacidosis improved his condition, the patient died in 2023 due to cerebral infarction.

**Conclusion:**

This case embodies the typical manifestations of mitochondrial diseases, emphasizing the urgency of prompt diagnosis and symptom management, which largely depends on effective genetic screening.

## 1 INTRODUCTION

Mitochondria function as powerpacks of the cell, providing energy for nearly all biological activities. Human mitochondrial DNA (mtDNA) exhibits a circular double-stranded structure containing 16,569 base pairs and 37 genes, which mainly encode proteins controlling the respiratory chain and energy metabolism [1]. Due to the lack of histone protection and effective DNA repair capacity, mtDNA evinces a high chance of mutation with maternal inheritance [2]. Currently, 425 pathogenic mtDNA mutations have been reported, among which the most well-known type is m. 3243 A>G, located on the *MT-TL1* gene [3]. Deletion or point mutation of mtDNA disrupts the enzymes or carriers necessary for mitochondrial oxidative metabolism, preventing glycogen and fatty acids from entering the mitochondria to generate ATP. This leads to energy deficiency and complex clinical symptoms, including myopathy (53%), epilepsy (50%), stroke-like episodes (48%), hearing loss (44%), dementia (27%), and diabetes (15%) [4]. Additionally, some mitochondrial diseases present as syndromes, such as mitochondrial encephalomyopathy associated with lactic acidosis and stroke-like episodes (MELAS) [5], chronic progressive external ophthalmoplegia [6], and Kearns-Sayre syndrome [7]. In terms of epidemiology, mitochondrial diabetes mellitus (MDM) affects up to 3% of diabetes patients. MDM is most commonly diagnosed in the age range of 35–40 years, whereas MELAS develops mainly during childhood. To date, studies on these conditions involve mainly case reports due to the low prevalence of the diseases. Meanwhile, the diversity of clinical features is likely to contribute to misdiagnosis and missed diagnosis. Even for experienced clinicians who consider the possibility of such conditions, a confirmatory diagnosis remains challenging due to heterogeneity of specimen collection and lack of screening techniques [8]. Here we report a case of a patient with both MELAS and MDM due to 3243 A>G mutation at the *MT-TL1* gene. The patient presented with diabetic symptoms for 5 years. Subsequently, cognitive manifestations appeared and worsened gradually. Insulin was administrated after genetic screening. Symptomatic treatment was carried out, which alleviated the condition substantially. However, the patient’s blood glucose remained poorly controlled, and he died of cerebral infarction during follow-up.

## 2 CASE PRESENTATION

A 36-year-old male was admitted on May 20, 2022, due to abdominal pain and nausea for 13 hours, accompanied by dry mouth, excessive thirst, and frequent urination. He showed cognitive impairment and unsteady gait due to cerebral infarction and brain atrophy.

The patient had been diagnosed with type 2 diabetes mellitus and was receiving treatment with metformin hydrochloride 0.5 g Tid and acarbose 50 mg for 5 years. Initially, his blood glucose was controlled satisfactorily. However, in 2019, he was hospitalized three times for epilepsy, diabetic ketoacidosis, and herpes zoster with glycated hemoglobin and fasting glucose levels reaching 8.5% and 10.33 mmol/L, respectively. The patient, due to a history of epilepsy for more than 10 years, was taking sodium valproate. With regard to family history, the patient’s mother was neither obese nor hearing-impaired, nor was her diabetes well controlled by metformin. The patient’s sister and son showed no signs of diabetes or hearing impairment.

**Fig. (1):**
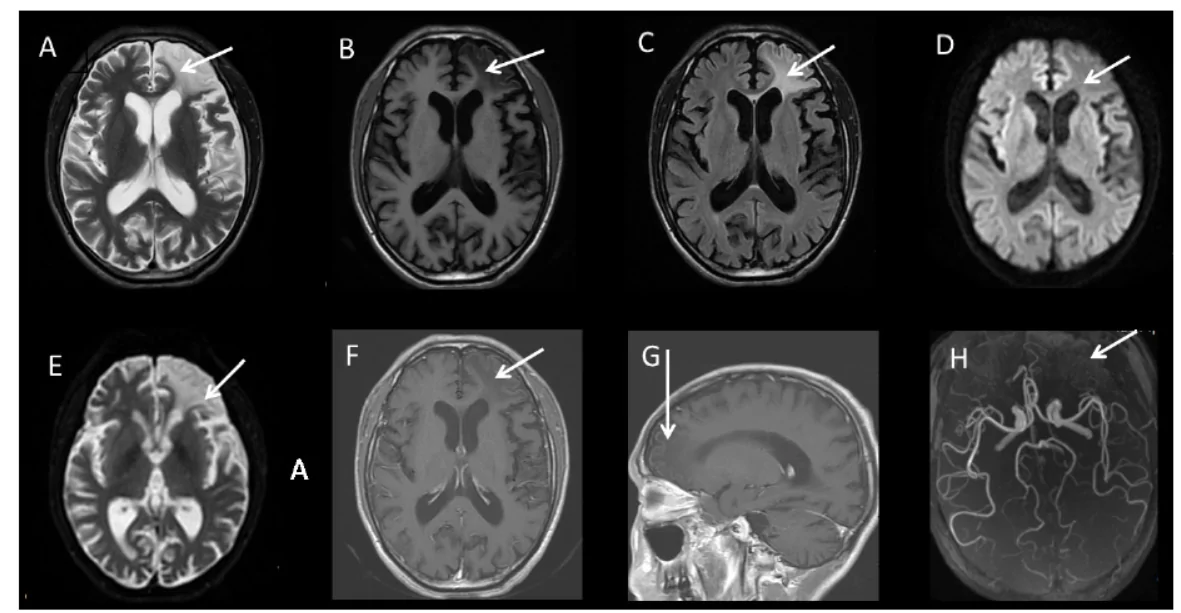
Representative images from cranial magnetic resonance imaging (MRI) of the patient. (**A**) T2-weighted imaging (T2WI); (**B**) T1-weighted imaging (T1WI); (**C**) T2 fluid-attenuated inversion recovery (T2-FLAIR); (**D**) diffusion-weighted imaging (DWI, 1000b); and (**E**) apparent diffusion coefficient (ADC) revealed pronounced atrophy and patchy hyperintensity zones in the left frontal lobe (white arrows), which showed no enhancement on contrast MRI (**F-H**) (white arrows). Magnetic resonance angiography (**H**) detected normal cerebral vessels.

The following parameters were recorded from general examination: heart rate of 112 beats/min, height of 160 cm, weight of 37.5 kg, and body mass index (BMI) of 14.65 kg/m^2^. Hearing loss was found on a rough check. The muscle strength of his limbs was grade 4. Blood tests indicated a glycated hemoglobin of 20.6%, β-hydroxybutyrate concentration of 5.4 mmol/L, random blood glucose of 37.35 mmol/L, total cholesterol of 6.37 mmol/L, low-density lipoprotein cholesterol of 4.04 mmol/L, fasting C-peptide concentration of 0.20 ng/L, postprandial 2-hour C-peptide of 0.96 ng/L, negativity for diabetes autoantibodies, pH value of 7.19, and lactate concentration of 3.0 mmol/L. T2-weighted imaging (T2WI), T1-weighted imaging (T1WI), T2-weighted fluid-attenuated inversion recovery (T2-FLAIR), diffusion-weighted imaging (DWI, 1000b), and apparent diffusion coefficient (ADC) calculation from cranial magnetic resonance imaging (MRI) revealed a patchy atrophy in the left frontal lobe, which was not intensified on contrast scanning. The remaining brain parenchyma was normal (Figs. **1A**-**G**). Magnetic resonance angiography (MRA) found no unusual cerebral vessels (Fig. **1H**). Electroencephalography (EEG) showed a basic rhythm of α waves at 8–12 Hz and 20–30 UV, with a few low-amplitude β waves at 14–20 Hz and θ waves at 4–7 Hz. The signals of the left and right cerebral hemispheres were roughly symmetrical under an acceptable modulation and amplitude. No abnormal waves were observed. Peripheral blood cell gene sequencing detected a mutation of m.3243A>G in the *MT-TL1* gene, with a ratio of 6.04%. Based on these findings, the diagnosis of MELAS and MDM with ketoacidosis was made without much challenge.

During hospitalization, insulin aspart 4 IU was administered before each meal, with additional doses of 3–5 IU based on blood glucose measurements. Additionally, glargine insulin 10 IU was given, with additional doses of 2–6 IU according to blood glucose measurements. Fluid replacement and symptomatic treatment were performed to correct ketoacidosis. On May 23, 2022, the blood pH value was maintained to 7.43, but the blood lactate concentration was still high at 5.2 mmol/L. Meanwhile, the patient experienced seizures with bilateral leftward eye gaze and tense limb muscles, which were alleviated by diazepam 10 mg and phenobarbital sodium 0.1 g given intravenously. The patient’s abdominal pain subsided following blood glucose reduction and ketoacidosis correction. Due to cognitive impairment, he could not cooperate with hearing tests. At discharge, his blood glucose level (capillary blood) remained abnormal, with fasting blood glucose ranging from 4.6 to 10.9 mmol/L and 2-hour postprandial from 10.0 to 20.6 mmol/L. There were no recurrent seizures. Discharge instructions included repaglinide 2 mg orally three times daily before meals, empagliflozin 10 mg orally once daily, linagliptin 5 mg orally once daily, and insulin glargine 8 IU subcutaneously at bedtime for diabetes control. Moreover, coenzyme Q10 capsules 10 mg orally three times daily and levetiracetam 0.5 g orally twice daily were prescribed to suppress his epilepsy.

Four months after discharge (September 25, 2022), the patient was readmitted for diabetic ketoacidosis and pneumonia without seizures. Blood tests revealed a pH of 7.34, lactate concentration of 1.6 mmol/L, glycated hemoglobin of 19.1%, random blood glucose of 32.27 mmol/L, and β-hydroxybutyrate concentration of 6.1 mmol/L. His cognitive ability improved compared to before. After infection control, blood glucose reduction, and ketoacidosis correction, he was discharged with instructions to take empagliflozin 10 mg orally once daily, linagliptin 5 mg orally three times daily, and insulin glargine 8 IU subcutaneously at bedtime for blood glucose management, along with coenzyme Q10 capsules 10 mg orally three times daily and levetiracetam 0.5 g orally twice daily. Follow-up *via* WeChat with the patient’s family revealed a remaining cognitive disorder and hyperglycemia. In 2023, the patient died of cerebral infarction at another hospital.

## 3 DISCUSSION

The 3243 A>G mutation of the mitochondrial *MT-TL1* gene induces complex clinical manifestations. In the present case, both MDM and MELAS occurred, with the former arising 5 years before admission and the latter occurring later. The patient was first treated with metformin, but was switched to insulin and other antidiabetic drugs after genetic screening. Symptomatic treatment was arranged, which mitigated the cognitive disorders effectively. However, the patient still suffered from hyperglycemia and died of cerebral infarction during follow-up.

MDM is associated with maternal inheritance of mutations in mtDNA, affecting only female offspring [9]. The onset of diabetes typically occurs before the age of 40 years, mostly during adulthood, but not during adolescence. Testing for islet autoantibodies may be negative or reveal low or high levels, indicating MDM to be partly due to β-cell damage [10]. Glucose metabolism is defective, which causes damage to multiple organs, inducing hearing loss, vision decline, kidney disturbance, myopathy, mitochondrial encephalomyopathy, and cardiomyopathy [9]. Most patients maintain a normal or slightly low body weight, with average BMI values being less than 20 kg/m^2^. Short stature appears in many cases, ascribed to dysfunction of the hypothalamic-pituitary axis [11]. Among MDM patients, 75% experience sensorineural hearing loss due to an ion imbalance or cell death in blood vessels [12]. The prevalence of late-stage renal failure remains very high and can even be the only manifestation subsequent to the m.3243A>G mutation [11]. Myopathy typically affects the proximal limb muscles, presenting as muscle cramps or weakness [12]. The incidence of neurological damage, such as the axonal sensory-motor neuropathy, ranges from 10% to 27% [13]. Psychiatric disorders include recurrent severe depression, schizophrenia, and a variety of phobias [14]. Gastrointestinal discomforts, especially constipation and pseudo-obstruction, relate to high levels of the m.3243A>G mutation in the gastric mucosa [15]. Among neurological disorders, epilepsy relates closely to mtDNA mutations and the consequent energy deficiency. The underlying mechanisms include diminished sodium and potassium ATP enzymes, reduced membrane potential, oxidative stress, apoptosis, and defective calcium hemostasis [16, 17].

The diagnosis of MELAS and MDM relies on family history, typical clinical symptoms, imaging examinations, muscle electrophysiology, pathology, and molecular genetic diagnosis [9]. Among these approaches, MRI offers the advantages of non-invasiveness, feasibility, and rapidity. The MRI findings typically include cerebellar and cerebral atrophy as well as abnormal signals in the cortex and subcortex, with different syndromes exhibiting distinct characteristics [5, 7, 8, 18]. Typical subcortical (basal ganglia, brainstem, and cerebellum) lesions present as symmetric long T1 and long T2 signals. Magnetic resonance spectroscopy (MRS) is effective because it reveals abnormal lactate peaks in brain tissues or cerebrospinal fluids, the levels of which correlate with the severity of clinical symptoms [19]. In our case, the patient developed diabetes at the age of 30 years, with classic diabetic symptoms, ketosis, and negative islet autoantibodies. Hearing loss was detected. His mother also suffered from diabetes, indicating maternal inheritance. His short stature, thin body shape, and persistent high lactate levels even after correction of acidosis were consistent with mitochondrial diseases. Neurological lesions were evident, presenting as epilepsy, cerebral infarction, and ataxia, with cranial MRI revealing destructive neuronal lesions. The patient tested negative for islet autoantibodies, but this was not a requirement for the diagnosis [20]. Comprehensive genetic testing identified mitochondrial gene mutations. Therefore, the patient was confirmed to have MELAS and MDM.

Genetic screening remains the decisive tool for the identification of mitochondrial diseases. Normally, the severity of the disorders depends on the extent of mutation, with a high level (>85%) inducing MELAS while a low level (<45%) inducing MDM. It has been reported that the mtDNA heteroplasmy ratio ranges between 1–45% in leukocytes and 10–45% in pancreatic β-cells [21]. However, individuals bearing 0.01–0.1% mtDNA heteroplasmy also develop diabetes, suggesting a high penetrance of this mutation [22]. In the present case, the rate of mtDNA heteroplasmy was 6.04%, but the patient experienced both MELAS and MDM. This, on the one hand, indicates the complexity of the rare pathologic condition. On the other hand, novel approaches may need to be established to raise the sensitivity and accuracy of the analysis [23].

Currently, the treatment of mitochondrial diseases emphasizes the avoidance of triggering conditions, such as infections and excessive fatigue [24]. Most patients maintain a normal or slightly low body weight, and thus, dietary restrictions are not recommended. Resistance and endurance training may help improve mitochondrial quality. In terms of pharmacological approaches, insulin but not metformin should be started as early as possible upon diagnosis, as the latter promotes the production of lactate [25]. Sulfonylureas, sodium-glucose cotransporter 2 inhibitors (SGLT2is), and glucagon-like peptide 1 receptor agonists (GLP-1RAs) are considered safer options [26, 27]. For mitochondrial-targeted medications, drugs that enhance electron transport chain activity, such as coenzyme Q10, along with idebenone, antioxidants, and metabolic co-factors, including citrulline and arginine, can be adopted. Cocktail therapy, which combines various antioxidants and co-factors, has been shown to effectively improve the conditions of patients [28]. Strategies for symptom management involve antiepileptic drugs, hearing aids, and cardiac pacemakers. Gene therapy and mitochondrial replacement are also topics of related research [29].

Genetic counseling for families with MDM is of great importance, as it helps asymptomatic patients to be diagnosed early and managed properly. In our case, the patient did not receive genetic screening at the onset of diabetes, and metformin was used for glycemic control, which first controlled his blood glucose well, but later became ineffective. This variation indicated a shift from an insulin-independent to insulin-dependent condition, which has been mentioned in other reports [8]. Meanwhile, the patient gradually developed hyperlactacidemia, meaning the glucose utilization declined further. Therefore, for young diabetes patients, the possibility of MDM should not be overlooked.

Metformin lowers blood glucose by inhibiting hepatic gluconeogenesis and increasing the utilization of glucose by peripheral tissues, which may restrain lactate metabolism under hypoxia or metabolic impairment and result in lactate accumulation. Most reports designate metformin as being unfit for the treatment of MDM; however, the beneficial effects of the drug on mitochondrial functions should still be recognized, as it reduces oxidative stress, deletes mutation frequency, attenuates mechanistic target of rapamycin (mTOR) and extracellular signal-regulated kinase (ERK) signaling, enhances autophagy, ameliorates inflammation, and abrogates NLRP3 inflammasomes [30].

In the later stages of the present case, insulin, fluid replacement, and symptomatic treatment were performed to correct ketoacidosis. Coenzyme Q10 was applied to enhance energy generation, complying with canonical approaches, which reduced the blood glucose levels and reversed ketoacidosis effectively. However, as mitochondrial dysfunction elicits intense oxidative stress and peroxidative damage, which may aggravate the manifestations of MDM and MELAS, antioxidant therapy should also be considered [31]. Some studies have observed the potency of chemical compounds, such as tryptolinamide and L-arginine, for relieving the energetic defects in MELAS [32, 33], but these were not applied in our case.

Although receiving targeted care, the patient suffered from a relapse soon and died of cerebral infarction during follow-up. This could be a consequence of mitochondrial dysfunction in cerebral endothelial cells (ECs). Insufficient energy supply may damage the integrity of ECs, leading to inflammation, oxidative stress, and the dysregulation of cerebral blood flow. Moreover, hyperglycemia and insulin resistance exacerbate the injury of cerebral arteries, which may accelerate the occurrence of infarction. Thus, for patients with MDM and/or MELAS, in addition to symptomatic management and supportive care, vascular protection approaches, such as anti-inflammatory and antioxidant therapies, could be beneficial. Even with the deficiency, the unfavorable outcome of this case highlights a limitation of current therapeutic strategies for mitochondrial diseases.

## 4 STUDY LIMITATION

In the management of the present case, genetic screening was not conducted until admission, before which metformin had been given for blood glucose control. Moreover, anti-inflammatory and antioxidant agents were not utilized to mitigate damage to tissues resulting from energy deficiency.

## CONCLUSION

In summary, timely diagnosis through a comprehensive analysis of clinical characteristics, physical examination, laboratory test results, imaging features, family medical history, and mitochondrial genetic screening is of critical importance for implementing targeted treatment and care for MELAS and MDM. At present, individualized and symptomatic treatments are still the first therapeutic choices.

## Data Availability

The data sets used and/or analysed during this study are available from the corresponding author upon request.

## LIST OF ABBREVIATIONS

ADC: Apparent Diffusion Coefficient
BMI: Body Mass Index
DWI: Diffusion-Weighted Imaging
EEG: Electroencephalography
GLP-1RAs: Glucagon-Like Peptide 1 Receptor Agonists
MDM: Mitochondrial Diabetes Mellitus
MRI: Magnetic Resonance Imaging
MRA: Magnetic Resonance Angiography
mtDNA: Mitochondrial DNA
MELAS: Mitochondrial Encephalomyopathy Associated with Lactic Acidosis and Stroke-Like Episodes
SGLT2is: Sodium-Glucose Cotransporter 2 Inhibitors
T2WI: T2-Weighted Imaging
T1WI: T1-Weighted Imaging
T2-FLAIR: T2-Weighted Fluid-Attenuated Inversion Recovery

## STANDARDS OF REPORTING

CARE guidelines were followed.
